# Microbial-host-isozyme: unveiling a new era in microbiome–host interaction

**DOI:** 10.1080/19490976.2023.2267185

**Published:** 2023-10-10

**Authors:** Lei Miao, Herbert Tilg, Ming-Hua Zheng

**Affiliations:** a Department of Gastroenterology, the Second Affiliated Hospital of Wenzhou Medical University, Wenzhou, Zhejiang, China; b Department of Internal Medicine, Gastroenterology, Hepatology, Endocrinology & Metabolism, Medical University Innsbruck, Innsbruck, Austria; c MAFLD Research Center, Department of Hepatology, The First Affiliated Hospital of Wenzhou Medical University, Wenzhou, China; d Department of laboratory, Key Laboratory of Diagnosis and Treatment for The Development of Chronic Liver Disease in Zhejiang Province, Wenzhou, Zhejiang, China

**Keywords:** Microbial-Host isozymes, metabolic diseases, metabolic diseases

## Abstract

Wang K. et al. introduced the concept of Microbial-Host isozymes (MHIs) and highlighted their role in mediating microbiota–host interactions. They identified bacterial-derived DPP4 as an isoenzyme affecting glucose tolerance and showed that host DPP4 inhibitors may not effectively target bacterial DPP4. They developed an MHI screen system, identifying 71 MHIs in healthy gut microbiota. Among them, DPP4 isozymes degrade GLP-1, explaining variable responses to sitagliptin. This breakthrough opens new avenues for metabolic disorder treatment. However, the complex nature of gut symbiotic bacteria requires further research to understand MHI mechanisms, regulatory roles, and interactions with the host. Precise interventions in gut microbiota offer personalized approaches to metabolic diseases.

We were impressed by the data presented by Wang K. et al. on developing an activity-based microbial-host isozymes (MHIs) screening system. The authors presented for the first time the concept of MHIs and emphasized the widespread presence of bacterial MHIs in the intestine. MHI is a kind of enzyme that could mediate interactions between microbiota and host, linking microbial enzyme activity with the host’s physiological functions.^1^ These MHIs can simulate host enzyme functions and participate in the occurrence and development of metabolic diseases (MD).^2,3^

First, the authors performed that bacterial-derived DPP4 is an isoenzyme that can enter intestinal tissue under intestinal barrier damage conditions, degrade host GLP-1, and affect glucose tolerance.

Second, the authors demonstrated that the host DPP4 inhibitor sitagliptin cannot effectively inhibit the activity of bacterial DPP4, which is an important reason for the large individual differences in the clinical response to sitagliptin.

Finally, the authors developed an activity-based MHI screening system to assess the enzyme activity of 110 enzymes related to human diseases in the human gut microbiota(Figure 1). The system consisted of an anaerobic bacteria culture system that could mimic the composition of human gut microbiota and 110 kinds of enzyme assays. Seventy-one MHIs were identified in the gut microbiota of healthy volunteers throng this system.^4–6^ The ten major systems of human are susceptible to metabolic disorders, often linked to deficiencies in metabolic enzymes, we categorize the 71 identified MHIs by different systems (Supplementary Table A1). Among the 71 MHIs, dipeptidyl peptidase 4 (DPP4) isozymes mainly produced by Bacteroides spp., could degrade active glucagon-like peptide-1 (GLP-1), affecting glucose tolerance and diabetes onset. This discovery helps to explain varied responses to the clinical drug sitagliptin.^1^

**Figure 1.**
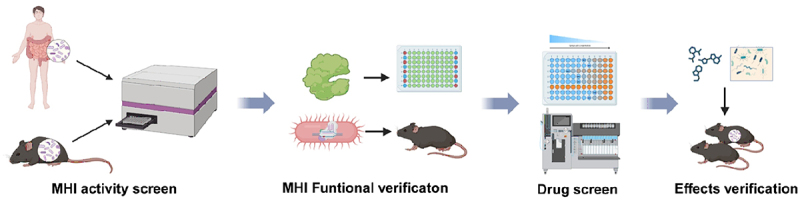
Microbial-host-isozyme mining and verification system.

The research team established a comprehensive MHIs mining and verification system, through four key phases. MHI activity screen initiates the identification of host-microbial isozyme interactions. MHI functional verification rigorously examines these interactions, unveiling MHI physiological significance. Drug screen explores MHI’s therapeutic potential, and screening compounds that modulate interactions to influence pathways. Finally, Effects verification substantiates compound impact on MHI, paving the way for targeted therapies. MHI’s mining and verification system represents a collaborative narrative, from discovery to effects verification.

This breakthrough demonstrates the potential of targeting MHI for addressing metabolic disorders, opening a novel avenue in gut microbiota research. However, due to the intricate taxonomic and evolutionary complexities of gut symbiotic bacteria, enzymes mirroring host functions frequently display reduced homology. This phenomenon restricts the utility of conventional sequence-based omics approaches such as metagenomics and metaproteomics in exploring the application of gut symbiotic microbial-host isozymes.^7^ MHIs still require further in-depth research to comprehensively understand and explore their mechanisms, regulatory mechanisms, and roles under different physiological conditions. Investigating the manifestations of MHI in various gut environments will shed light on the interactions between gut microbiota, MHI, and the host. Exploring the functionality of gut microbial enzymes and intervention strategies is crucial. This demands interdisciplinary collaboration and more extensive research efforts to unveil new insights and directions for the treatment and prevention of metabolic disorders. Precision research and treatment in the field of gut microbiota hold paramount significance, offering potential avenues for personalized interventions in metabolic diseases.

## Supplementary Material

Supplemental Material
