# Supplementary material for: Microbial-host-isozyme: unveiling a new era in microbiome–host interaction
Source: Gut Microbes. 2023 Oct 10;15(2):2267185. doi: 10.1080/19490976.2023.2267185 (PMC10566401; doi:10.1080/19490976.2023.2267185)
Supplement: Supplemental Material [file KGMI_A_2267185_SM2430.docx]

**Supplementary Table 1**.  **71 identified microbial-host isozymes in ten major systems of human body.**

| **Respiratory System** | **Cardiovascular System** | **Digestive System** | **Endocrine System** | **Nervous System** | | **Immune**  **System** | **Urinary System** | **Skin**  **System** | **Reproductive System** | **Skeletal and Muscular System** |
| --- | --- | --- | --- | --- | --- | --- | --- | --- | --- | --- |
| Amino-acid N-acetyltransferase (EC:2.3.1.1) | Tyrosine aminotransferase (EC:2.6.1.5) | Biliverdin reductase (EC:1.3.1.24) | Dipeptidyl peptidase 4 (EC:3.4.14.5) | | Choline O-acetyltransferase (EC:2.3.1.6) | Purine-nucleoside phosphorylase (EC:2.4.2.1) | Cystathionine beta-synthase (EC:4.2.1.22) | Farnesyl diphosphate synthase  (EC:2.5.1.1) | Aromatase (EC:1.14.14.14) | Creatine kinase M-type(EC:2.7.3.2) |
| Phosphoserine transaminase (EC:2.6.1.52) | Membrane alanyl aminopeptidase (EC:3.4.11.2) | Amine oxidase (EC:1.4.3.4) | Phosphoglycerate dehydrogenase (EC:1.1.1.95) | | Nicotinamide mononucleotide adenylyltransferase (EC:2.7.7.1) | Granzyme B (EC:3.4.21.79) | Xanthine dehydrogenase (EC:1.17.1.4) | Tyrosinase  (EC:1. 14. 18. 1) | Arylsulfatase A(EC:3.1.6.8) |  |
|  | Arylesterase (EC:3.1.1.2) | Carboxylesterase (EC:3.1.1.1) | Triacylglycerol lipase (EC:3.1.1.3) | | Kynureninase (EC:3.7.1.3) | Hyaluronoglucosaminidase (EC:3.2.1.35) | Sulfite oxidase(EC:1.8.3.1) | Guanine deaminase  (EC:3.5.4.3) | 3-oxo-5-alpha-steroid 4-dehydrogenase 1(EC:1.3.1.22) |  |
|  | UDP-glucose 6-dehydrogenase (EC:1.1.1.22) | Carbonyl reductase (EC:1.1.1.184) | Alpha-galactosidase (EC:3.2.1.22) | | Memapsin 2 (EC:3.4.23.46) | Caspase-1(EC:3.4.22.36) |  |  |  |  |
|  | Carboxypeptidase U (EC:3.4.17.20) | Adenosine deaminase (EC:3.5.4.4) | 7dehydrocholesterol reductase (EC:1.3.1.21) | | Acetylcholinesterase (EC:3.1.1.7) | Glucose-6-phosphate isomerase(EC:5.3.1.9) |  |  |  |  |
|  | Arachidonate 5-lipoxygenase (EC:1.13.11.34) | Cathepsin B (EC:3.4.22.1) | Nicotinamide N-methyltransferase (EC:2.1.1.1) | | GMP reductase (EC:1.7.1.7) | Inosine-5'-monophosphate dehydrogenase 1( EC:1.1.1.205) |  |  |  |  |
|  | Gamma-glutamyl hydrolase  (EC:3.4.19.9) | Uridine phosphorylase (EC:2.4.2.3) | Methionine aminopeptidase (EC:3.4.11.18) | | Tryptophan 5-monooxygenas (EC:1.14.16.4) | CTP synthase 1(EC:6.3.4.2) |  |  |  |  |
|  | Soluble epoxide hydrolase  (EC:3.3.2.10) | Carbonic anhydrase (EC:4.2.1.1) | 17beta-estradiol 17-dehydrogenase (EC:1.1.1.62) | | Dihydropyrimidinase (EC:3.5.2.2) | Acetyl-CoA carboxylase 1(EC:6.4.1.2) |  |  |  |  |
|  | L-asparaginase (EC:3.5.1.1) | Ornithine aminotransferase (EC:2.6.1.13) | Gamma-glutamyltransferase (EC:2.3.2.2) | | Glutamine synthetase (EC:6.3.1.2) | Histidine decarboxylase  (EC:4.1.1.22) |  |  |  |  |
|  | Aldehyde dehydrogenase (EC:1.2.1.3) | α-Amylase (EC:3.2.1.1) | GTP cyclohydrolase 1 (EC:3.5.4.16) | | Tyrosine 3-monooxygenase (EC:1.14.16.2) |  |  |  |  |  |
|  | Ceramide glucosyltransferase (EC:2.4.1.80) | Thymidine phosphorylase (EC:2.4.2.4) | L-iditol 2-dehydrogenase (EC:1.1.1.14) | | Prolyl endopeptidase  (EC:3.4.21.26) |  |  |  |  |  |
|  | Glucose-6-phosphate dehydrogenase (EC:1.1.1.49) | 15-hydroxyprostaglandin dehydrogenase (EC:1.1.1.141) | L-xylulose reductase (EC:1.1.1.10) | | Adenylyl cyclase 1(EC:4.6.1.1) |  |  |  |  |  |
|  | Adenosylhomocysteinase (EC:3.13.2.1) | Trypsin (EC:3.4.21.4) | Aldose reductase (EC:1.1.1.21) | | Arginase-2(EC:3.5.3.1) |  |  |  |  |  |
|  | Thioredoxin-dependent peroxiredoxin  (EC 1.11.1.24) | Delta-aminolevulinic acid dehydratase(EC:4.2.1.24) | Proline dehydrogenase (EC:1.5.5.2) | | Beta-galactosidase(EC:3.2.1.23) |  |  |  |  |  |
|  | L-lactate dehydrogenase (EC:1.1.1.27) | Alcohol dehydrogenase (EC:1.1.1.1) | Insulin-degrading enzyme(EC:3.4.24.56) | | Inositol monophosphatase 1(EC:3.1.3.25) |  |  |  |  |  |
|  | Nitric oxide synthase(EC:1.14.13.39) | Glutathione-disulfide reductase (EC:1.8.1.7) | Neutral alpha-glucosidase C(EC:3.2.1.20) | | Peptidyl-prolyl cis-trans isomerase B(EC:5.2.1.8) |  |  |  |  |  |
|  | Dihydrofolate reductase(EC:1.5.1.3) | Glycine N-acyltransferase (EC:2.3.1.13) | Fructose-1,6-bisphosphatase 1(EC:3.1.3.11) | | Protein disulfide-isomerase (EC:5.3.4.1) |  |  |  |  |  |
|  | 3-hydroxy-3-methyl glutaryl coenzyme A reductase(EC:1.1.1.34) | 5'-nucleotidase (EC:3.1.3.5) | 3 beta-hydroxysteroid dehydrogenase type 7(EC:1.1.1.-) | | Thymidylate synthase(EC:2.1.1.45) |  |  |  |  |  |
|  | Acyl-CoA synthetase 1(EC:6.2.1.3) | Protein farnesyltransferase (EC: 2.5.1.58) |  | | Lactoylglutathione lyase(EC:4.4.1.5) |  |  |  |  |  |
|  | Phospholipase A2(EC:3.1.1.4) | Intestinal-type alkaline phosphatase(EC:3.1.3.1) |  | | Dihydropyrimidine dehydrogenase(EC:1.3.1.2) |  |  |  |  |  |
|  | Cytosolic beta-glucosidase(EC:3.2.1.21) | Glutamate dehydrogenase 2(EC:1.4.1.3) |  | | Catalase (EC:1.11.1.6) |  |  |  |  |  |
|  | Prolyl 3-hydroxylase 1(EC:1.14.11.7) | L-glutaminase(EC:3.5.1.2) |  | |  |  |  |  |  |  |
|  | Chitotriosidase-1(EC:3.2.1.14) | Glutathione S-transferase A1(EC:2.5.1.18) |  | |  |  |  |  |  |  |
|  | Angiotensin-converting enzyme(EC:3.4.15.1) |  |  | |  |  |  |  |  |  |
|  | Carbamoyl-phosphate synthase(EC:6.3.4.16) |  |  | |  |  |  |  |  |  |
|  | Malate dehydrogenase(EC:1.1.1.37) | . |  | |  |  |  |  |  |  |
|  | Methylenetetrahydrofolate reductase(EC:1.5.1.20) |  |  | |  |  |  |  |  |  |
